# Comparison of Rheological Properties of Healthy versus Dupuytren Fibroblasts When Treated with a Cell Contraction Inhibitor by Atomic Force Microscope

**DOI:** 10.3390/ijms24032043

**Published:** 2023-01-20

**Authors:** Sandra Pérez-Domínguez, Javier López-Alonso, Frank Lafont, Manfred Radmacher

**Affiliations:** 1 Institute of Biophysics, University of Bremen, 28359 Bremen, Germany; 2 University of Lille, CNRS, INSERM, CHU Lille, Institute Pasteur Lille, U1019-UMR 9017-CIIL-Center for Infection and Immunity of Lille, 59021 Lille, France

**Keywords:** Dupuytren’s disease, fibroblast, atomic force microscopy, rheology, ML-7, inhibition

## Abstract

Mechanical properties of healthy and Dupuytren fibroblasts were investigated by atomic force microscopy (AFM). In addition to standard force curves, rheological properties were assessed using an oscillatory testing methodology, in which the frequency was swept from 1 Hz to 1 kHz, and data were analyzed using the structural damping model. Dupuytren fibroblasts showed larger apparent Young’s modulus values than healthy ones, which is in agreement with previous results. Moreover, cell mechanics were compared before and after ML-7 treatment, which is a myosin light chain kinase inhibitor (MLCK) that reduces myosin activity and hence cell contraction. We employed two different concentrations of ML-7 inhibitor and could observe distinct cell reactions. At 1 µM, healthy and scar fibroblasts did not show measurable changes in stiffness, but Dupuytren fibroblasts displayed a softening and recovery after some time. When increasing ML-7 concentration (3 µM), the majority of cells reacted, Dupuytren fibroblasts were the most susceptible, not being able to recover from the drug and dying. These results suggested that ML-7 is a potent inhibitor for MLCK and that myosin II is essential for cytoskeleton stabilization and cell survival.

## 1. Introduction

Dupuytren’s disease is a fibromatosis of the connective tissue of the palm, which—in the worst case—leads to bending of the hand and/or one or more fingers hampering regular hand activities. Few non-invasive strategies have been used to release tension, such as needle acupuncture and collagenase injection [1,2]; however, in most cases, the contraction recurs. In the case of advanced stages of the disease, surgery is the most common solution [3]. In patients who suffer from Dupuytren’s disease, the formation of nodules and cords in the palmar fascia is observed, which is caused by aggregation of proliferated fibroblasts that adhere to fibrin. The palmar fascia is made of bands of connective tissue that are mainly composed of type I collagen. Between the collagen fibers, rows of fibroblasts can be found, which secrete and deposit extracellular matrix (ECM) components. In wound healing, healthy fibroblasts undergo a phenotypic change into myofibroblasts expressing α-smooth muscle actin (α-SMA). The expression of α-SMA along with fibronectin ectodomain A is activated by the latent transforming growth factor β-1 protein (TGFβ-1) that is present in the ECM. Furthermore, in the palmar fascia of patients with Dupuytren’s disease, an increase in collagen III/I ratio was found together with an increased differentiation of myofibroblasts leading to an increment of hand contraction [4,5,6]. Similarities between myofibroblasts and smooth muscle (SM) cells were suggested in initial studies. Although both cell types present α-SMA, SM cells contraction is rapid and short in duration, while myofibroblast contraction is rather long-lasting and results in permanent contraction [6]. In wound healing, a few cytokines and growth factors (interleukins, TGFβ-1, etc) are released that have been involved in the stimulation of myofibroblast transition. Previous experiments suggested that exposing healthy fibroblasts to TGFβ-1 leads to differentiation into a myofibroblast phenotype [7,8,9]. Moreover, lately a comparison between healthy and Dupuytren fibroblasts exposed to TGFβ-1 showed an increase in α-SMA expression in the case of Dupuytren fibroblasts but not in healthy fibroblasts, which corroborates the myofibroblast phenotype assignment to Dupuytren cells [7].

Many studies have tried to develop a strategy to revert myofibroblast to fibroblast differentiation. Unfortunately, not many have been successful. Interferon-γ (IFN-γ) has been reported as a possible therapeutic agent for contractile diseases in clinical trials and in vitro studies [10,11,12]. However, late results suggested that IFN-γ inhibits myofibroblast generation and downregulates α-SMA production in TGFβ-1-induced myofibroblasts, but it does not produce the same effect on well-differentiated myofibroblasts [13,14].

ML-7 is a specific inhibitor of myosin light chain kinase (MLCK), a Ca^2+^/calmodulin-dependent enzyme that is essential for the activation of myosin II and cell contraction [15]. ML-7 reduces the phosphorylation of the myosin light chain (regulatory chain), resulting in the inability of the myosin head to change to the optimal conformation to bind to actin filaments, thus reducing actin–myosin interaction and contraction [16]. ML-7 is a reversible ATP-competitive inhibitor of MLCK that has been assessed as a potential chemotherapeutic drug for many diseases, such as prostate and mammary cancer [17,18], atherosclerosis [19], etc. A competitive inhibition occurs when the inhibitor molecule binds to the enzyme and blocks its activity. The inhibitor can bind either to the enzyme’s active site (competing with the enzyme-substrate natural binding) or to another site on the enzyme such that the enzyme’s activity is modified. However, as the inhibition is reversible, the inhibitor-enzyme interaction has a limited time life; therefore, the inhibitor will leave the enzyme allowing it to resume its regular enzymatic activity. In many diseases, such as pancreatitis, respiratory, cardiovascular and inflammatory bowel diseases, an abnormal expression of MLCK has been observed. Some works used ML-7 and analogs to study its potential beneficial effects on the previously mentioned disorders [20]. A high concentration of ML-7 produced a rounding up of Panc1 cells and a marked reduction in the number of stress fibers [21]. In total, 10 µM ML-7 was added to 3T3 and NRK cells, and they stopped migration, retracted their lamellipodia and softened by a factor of 3 in peripheral and nuclear regions. Besides, a concentration-dependent effect on the cells was observed [22].

In this study, AFM rheological experiments were designed to assess cell behavior. Rheology is the study of the flow and deformation of matter and takes into account the interplay between force, deformation and time. In rheology, often using oscillatory testing, the elastic modulus is represented as the storage modulus (E′) that refers to the stored energy in each oscillatory cycle and the viscous modulus, the loss modulus (E″) that measures the energy dissipated during one cycle as heat. The loss tangent (the ratio between loss and storage modulus) is taken as an indicator of the degree of solid- or liquid-like behavior. It can also be visualized as the tangent of the phase angle (δ) between the excitation (applied oscillatory force) and the response of the sample (indentation). The phase angle is the phase difference between stress and strain, and it will have a value equal to 0° for a pure elastic, 90° for a pure viscous and somewhat in the middle for viscoelastic materials.

AFM was initially developed as an imaging technique for hard samples; Lekka et al. were the first to employ the AFM as a diagnostic tool to discriminate between healthy and cancerous cells [23]. Nowadays, the use of AFM for measuring biological samples is widely spread; in particular, cell mechanics has become a promising approach to assess diseases at single-cell and tissue levels. Our AFM experimental setup for measuring cell rheological properties is based on oscillatory testing methodology, in which the cantilever is oscillated at constant amplitude, varying the frequency up to 1 kHz. Thanks to this AFM scheme called “sweep frequency”, storage and loss modulus, as well as loss tangent, could be computed.

This study sought the following two aims: (1) investigate and evaluate the rheological properties of three human primary cell types from Dupuytren’s disease. Due to the different phenotypes expressed by the cells, distinct rheological behaviors were expected. (2) Assess the effect of ML-7 as a cell contraction inhibitor and potential drug for reverting myofibroblast transition, tracking cell mechanical properties over time. We used 2 different concentrations (1 and 3 µM) in order to produce a significant effect while at the same time allowing the cells to recover.

## 2. Results

### 2.1. Rheological Characterization

In this work, three different types of fibroblasts from the palm of the same patient were investigated. We measured the rheological properties of healthy, scar and Dupuytren fibroblasts and searched for differences in cell behavior after the addition of a MLCK inhibitor. By using AFM conventional force curves and sweep frequency data (Figures S1–S3), we studied the viscoelastic response of the above-mentioned cells before and after ML-7 inhibitor addition in order to follow the cell mechanical changes over time. Firstly, the mechanical properties of these cells seeded on bare plastic Petri dishes were investigated using PFQNM-LC-A-CAL cantilevers. These cantilevers are useful for this type of experiments because although they are stiffer than cantilevers usually used for cell mechanics, such as MLCTs, they have a fairly large tip height and a large resonance frequency that makes them suitable for oscillatory measurements at high frequencies.

**Figure 1:**
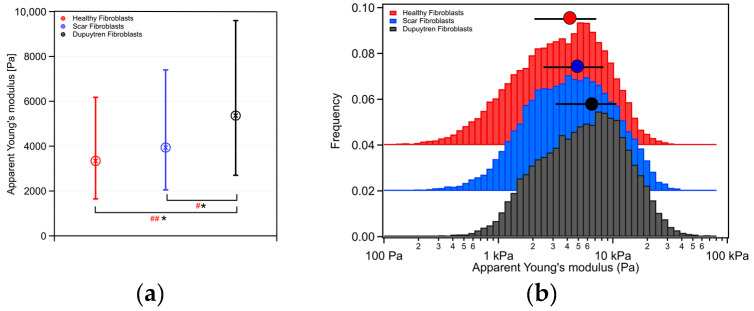
(**a**) Box plot of apparent Young’s modulus with the median and 25/75 percentiles for each fibroblast with Wilcoxon signed rank test significant difference: * indicating *p* < 0.01 and Cohen’s d test with # indicating 0.2 < d < 0.5 and ## indicating 0.5 < d < 1.0. (**b**) Histogram distribution of the apparent Young’s modulus for each fibroblast together with the median and 25/75 percentiles above each histogram (*n* = 90). Healthy fibroblasts are displayed in red, scar in blue and Dupuytren fibroblasts in black.

The 3D images of the three fibroblast types can be seen in Figure S4. Dupuytren fibroblasts showed larger apparent Young’s modulus values in comparison to scar and healthy fibroblasts. Moreover, scar fibroblasts presented slightly larger values than their healthy counterparts (Figure 1). A Wilcoxon signed-rank test and Cohen’s d statistical analysis were performed on apparent Young’s modulus, and statistically significant differences between healthy and Dupuytren and between scar and Dupuytren fibroblasts were found. Cohen’s d test suggested a medium-size effect between scar and Dupuytren fibroblasts and a large-size effect between healthy and Dupuytren fibroblasts.

**Figure 2:**
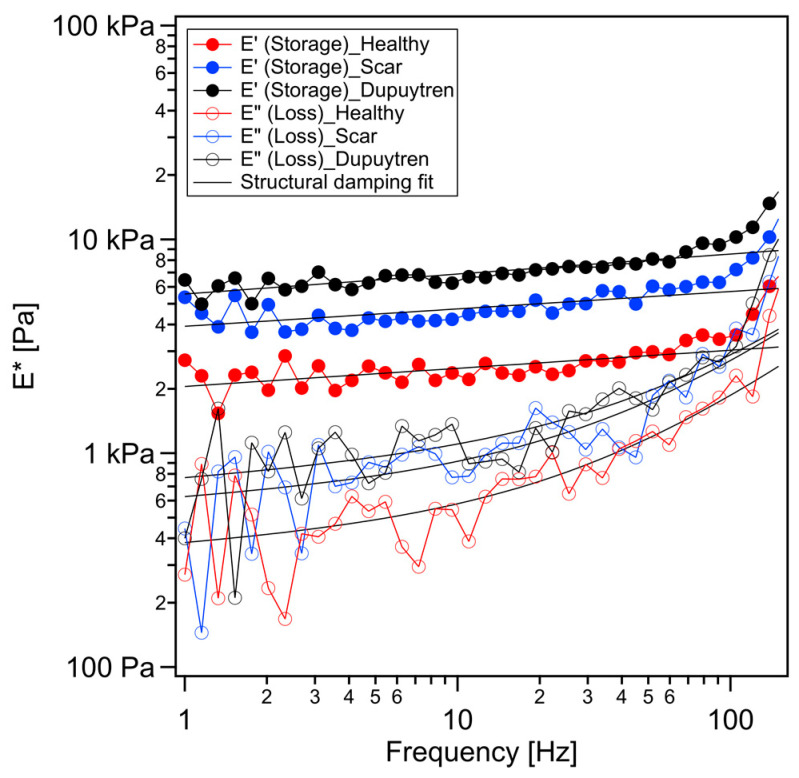
Frequency dependence of the storage (filled symbols) and loss moduli (open symbols) of healthy fibroblasts (red), scar fibroblasts (blue) and Dupuytren fibroblasts (black). Solid black lines are the fit of the power-law structural damping model (*n* = 90). Data shown are corrected for the viscous drag.

Note that a hysteresis between approach and retract force curves was visible due to cell viscosity (Figure S1). Accordingly, the values of the elastic modulus extracted from the approach and the retract curves using Hertz models differ significantly when investigating cells. Hence, this is clear evidence of how important the viscous contribution of cells, which cannot be easily separated from the elastic one in conventional force curves, is. To solve this issue, we used the sweep frequency methodology to measure the elastic and viscous response of the cell samples. Briefly, after approaching the tip towards the cell up to a deflection value defined by the trigger threshold, the z-piezo motion was stopped for 1 s. During this time, the stress applied during the approach ramp of the cell relaxed, and we applied a sinusoidal modulation with increasing frequency while still in contact with the cell. The frequency was swept from 1 Hz to 1 kHz, although the later analysis was performed only from 1 Hz up to 100 Hz due to piezo response limitations. When the modulation finished, the cantilever was retracted as in a conventional force curve (Figures S2 and S3). We used the power-law structural damping model to analyze the elastic and viscous contribution of cells separately. The storage modulus E′ of healthy fibroblasts was 3.26 kPa at the lowest frequency applied (1 Hz) and increased linearly on a log-log scale (Figure 2), i.e., it actually followed a power law. The loss modulus E″ was approximately 6-fold smaller than E′: 0.47 kPa. Both storage and loss moduli displayed similar frequency dependence up to 10 Hz. Nevertheless, E″ showed a more marked frequency dependence at higher frequencies, which is to a large extent due to the hydrodynamic interaction of the cantilever with the liquid medium [24] (Figure 2). For this reason, a viscous drag correction was applied, subtracting cantilever-medium viscous contribution. Scar and Dupuytren fibroblasts showed a similar trend of storage and loss moduli over frequency (Figure 2).

**Figure 3:**
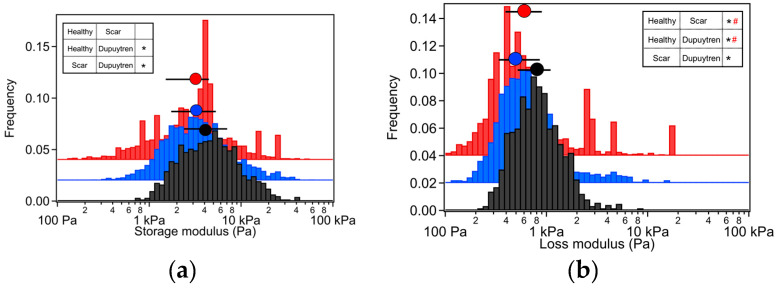
Histogram representation of the (**a**) storage and (**b**) loss modulus at 1 Hz for each fibroblast type together with the median and 25/75 percentiles above each histogram distribution. Healthy fibroblasts are in red, scar fibroblasts in blue and Dupuytren fibroblasts in black. Wilcoxon signed-rank test showing significant differences among all cell types are indicated in the table (*; *p* < 0.01) and Cohen’s d test with # indicating 0.2 < d < 0.5 (*n* = 90).

**Figure 4:**
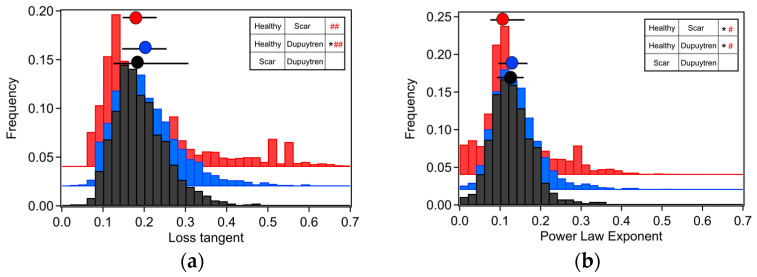
Histogram representation of the (**a**) loss tangent at 1 Hz and (**b**) power-law exponent for each fibroblast type together with the median and 25/75 percentiles above each histogram distribution. Healthy fibroblasts are in red, scar fibroblasts in blue and Dupuytren fibroblasts in black. Wilcoxon signed-rank test showing significant differences among all cell types are indicated in the table (*; *p* < 0.01) and Cohen’s d test with # indicating 0.2 < d < 0.5 and ## indicating 0.5 < d < 1.0 (*n* = 90).

Dupuytren fibroblasts displayed the highest values in almost all moduli (Table 1). The loss tangent and the power-law exponent of the three cell types presented very close values to each other. Significant differences among all cell types were found for all rheological parameters (Figure 3 and Figure 4). Both storage and loss moduli showed a similar trend as the apparent Young’s modulus, which could also be seen in the respective histograms (Figure 1b and Figure 3). The loss tangent at 1 Hz presented a barely significant difference among the cell types, the same as the power-law exponent (Figure 4). E_0_, which is a scale factor for the storage and loss moduli, varied depending on the cell type. Healthy and scar fibroblasts presented similar values (healthy: 3308 Pa and scar: 3077 Pa); nevertheless, Dupuytren fibroblasts showed an increase going up to 4259 Pa. The Newtonian viscous term (μ) was rather small, and it increased from healthy to Dupuytren fibroblasts (7 to 13 Pa·s, respectively).

### 2.2. Cytoskeleton Inhibition

The effect of ML-7 on the three different cell types was observed by video microscopy over a period of 2–3 h. Different concentrations of the inhibitor were added to the cells to decide which concentration was optimal to produce a significant change in cell mechanics but, at the same time, let the cells recover again.

**Figure 5:**
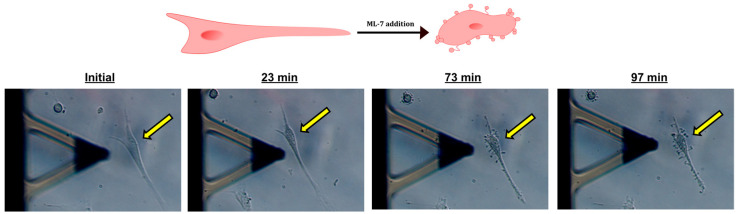
Effect of ML-7 addition on fibroblast morphology. Example of a scar fibroblast before and after the addition of 3 µM ML-7. Yellow arrow shows cell position. Cell contraction as well as bleb formation are visible.

With the help of time-lapse videos, the optimal concentration of ML-7 to observe a sufficient cell mechanical change was determined. In total, 1 and 3 µM ML-7 were sufficient to produce visual effects on the cells showing contraction and changes in morphology (Figure 5 and videos in Supplementary Materials). Mechanical properties of the three cell types were measured using the AFM before and after the addition of the inhibitor. We selected 5–6 cells in each experiment and focused on tracking the changes in stiffness over time in these 5–6 cells. Cell mechanics were probed twice before and 4–5 times after adding ML-7 inhibitor, spending a maximum time of 2–3 h per experiment. Consecutive measurements were performed every 15 or 30 min.

After adding 1 or 3 µM ML-7, we observed differences in cell mechanical behavior. Healthy and scar fibroblasts did not show a clear response after adding 1 µM; however, Dupuytren fibroblasts seemed to be more sensitive to the drug and experienced a decrease in apparent Young’s modulus right after the ML-7 addition (from 2 kPa to 0.2 kPa); after a while, they started to recover showing a slight increase in apparent Young’s modulus values (from 0.2 kPa up to 0.5 kPa) (Figure S5).

**Figure 6:**
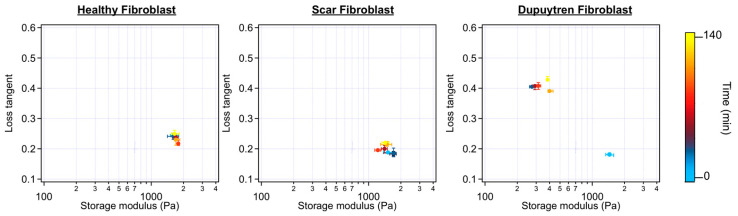
Scatterplot of the loss tangent versus storage modulus at 1 Hz before and after 1 µM ML-7 addition. The color bar indicates the time after addition (*n* = 15).

**Figure 7:**
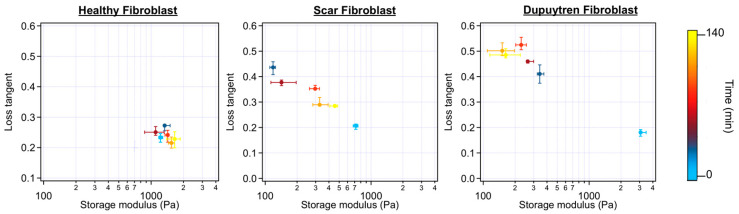
Scatterplot of the loss tangent versus storage modulus at 1 Hz before and after 3 µM ML-7 addition. The color bar indicates the time after addition (*n* = 20).

We also recorded the rheological parameters during these experiments, and the storage and loss moduli over the frequency of each fibroblast can be seen in Figure S6. Initial E* values (cell before ML-7 treatment), together with two intermediate times after a 1 µM ML-7 addition, were plotted. Healthy and scar fibroblasts did not show E* changes over frequency after inhibition; however, Dupuytren fibroblasts displayed a decrease in both moduli after ML-7 addition. A scatterplot of the loss tangent versus storage modulus at 1 Hz can be seen in Figure 6 and Figure 7. Each colored dot represents one cell measurement at a specific time point where the change towards lighter colors (from light blue-initial-, to yellow-last measurement-) corresponds to the time course during the experiment. In Figure 6 (Dupuytren fibroblast), the storage modulus followed the same trend as the apparent Young’s modulus (Figure S5), it decreased after ML-7 addition and it developed towards slightly higher values over time. The opposite trend was observed for the loss tangent, which increased after ML-7 addition. These results can be understood as a decrease in the elastic over the viscous elements due to the disruption of the cytoskeleton network caused by the inhibitor. Otherwise, when exposing the cells to 3 µM of ML-7, they showed larger changes (Figure 7). A good percentage of healthy and scar fibroblasts still did not show any visible effect (Table 2); however, others reacted to the inhibitor with a decrease in stiffness and either recovered over time or died (Figure S7).

An example of a force curve demonstrating the change in mechanics (slope) after 3 µM ML-7 can be found in Figure S8. In panel B, which is an example of a scar fibroblast, a decrease in slope was visible after 36 min of 3 µM ML-7 addition, which increased again after 118 min thanks to cell recovery. It can be noted how the hysteresis between approach and retract curves also increased over time in panel C owing to a decrease in cell elasticity. The frequency dependence of storage and loss moduli was shown for the three cell types after 3 µM ML-7 (Figure S9). The example of healthy fibroblasts did not present changes in E* after inhibitor addition; however, scar fibroblasts presented a decrease in both storage and loss moduli immediately after ML-7 addition but recovered after about 120 min. Dupuytren fibroblasts E* over frequency displayed a continuous decrease over time after ML-7 addition. Dupuytren fibroblasts, as already seen at lower ML-7 concentrations, either reacted more drastically to the drug, and many cells died or responded with a contraction and decrease in elastic modulus with recovery over time (Figure 7). Although the most representable data were shown in Figure 6 and Figure 7, not all fibroblasts from the same cell type reacted similarly. There was always a percentage of cells that died, recovered or did not respond to the ML-7 inhibitor regardless of cell type and inhibitor concentration (Table 2). Note that all rheological parameters of each fibroblast when treated with 1 and 3 µM ML-7 can be found in Figures S10–S15.

**Figure 8:**
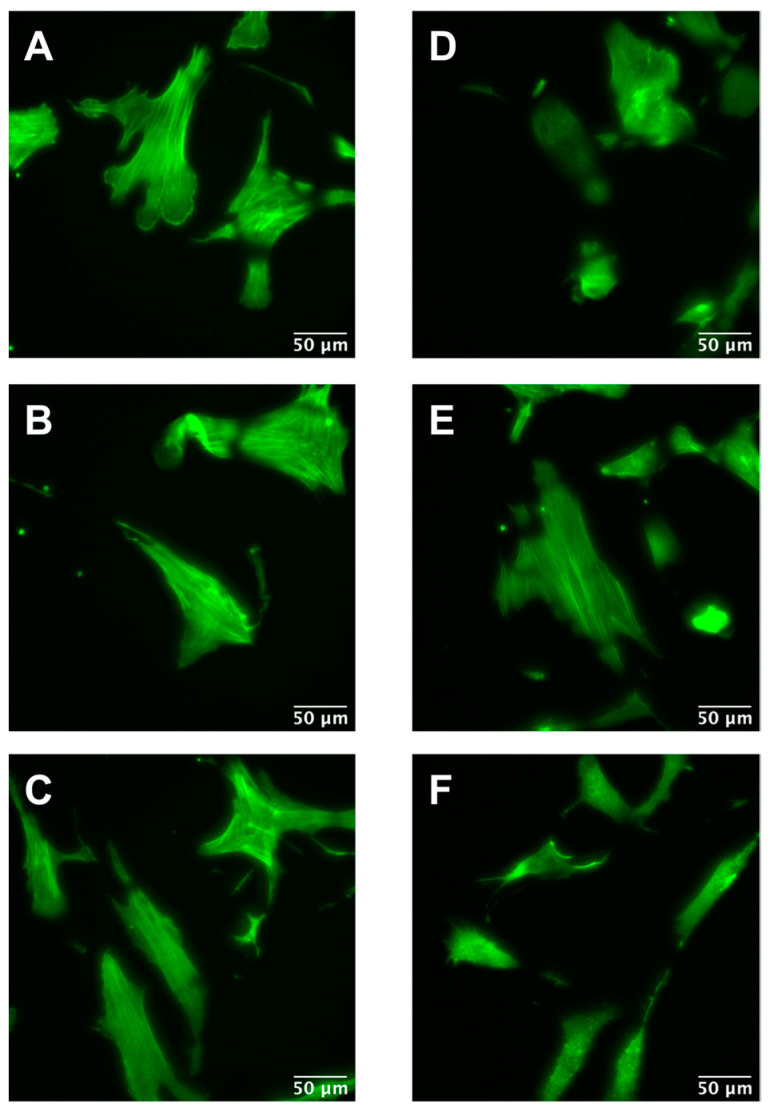
Fluorescence images of (**A**) healthy, (**B**) scar and (**C**) Dupuytren fibroblasts in normal conditions (no inhibitor); (**D**) healthy, (**E**) scar and (**F**) Dupuytren fibroblasts 20 min after the addition of 3 µM ML-7. Actin fibers are labeled in green. Control measurements adding DMSO at the same contraction as ML-7 can be found in Figures S16–S17. AFM measurements of Dupuytren fibroblasts over time without drug treatment can be observed in Figure S18.

To disentangle what happened to the cells in terms of the cytoskeleton network, actin fibers of the three cell types were labeled in all conditions (with and without ML-7 addition) (Figure 8). Cells affected by the inhibitor changed their shape (from elongated to more roundish due to a reduction in contractility) as well as actin fibers broke. In Figure 8F (Dupuytren fibroblasts treated with ML-7), some cells formed green dots in their interior along with blebs in the cell membrane, which could be due to cytoskeleton disruption (actin fibers break in small pieces) and cortex rupture. The bleb formation is an indicator of apoptosis initiation.

## 3. Discussion

### 3.1. Mechanical and Rheological Properties

Cells are living samples that are complicated to study and characterize. In most (AFM) cell mechanical studies, force curves are usually analyzed only in terms of Young’s modulus, which describes the mechanical behavior of elastic samples. More precisely, the widely used Hertz model quantifies the relationship between stress and strain only in the linear elastic regime of a material. Cells are much more complex materials: they are viscoelastic materials, i.e., they combine properties of viscous liquids and elastic solids. Purely elastic solids obey Hooke’s law, and when a stress is applied, an instantaneous strain appears, and on the removal of the stress, the strain reverts to zero. Viscous liquids are described to have a constant velocity flow and energy dissipation while a constant shear force is applied. In biophysics cell elasticity is understood in terms of cytoskeleton dynamics, where the number of crosslinks, rearrangements and deformation and stress (contractile forces) will affect the elastic properties of cells. The viscosity of cells is much more complicated to attribute, but it can be understood as the internal friction that occurs when all components in a flowing liquid are forced to slide along each other. For acquiring cells’ elastic and viscous properties, the AFM sweep frequency scheme was employed, which allows the separation of elastic and viscous components using an appropriate model. In the above graphs, the apparent Young’s modulus and the storage modulus displayed similar distributions, in which healthy fibroblasts presented the lowest values and Dupuytren fibroblasts the highest, which can be attributed to the changes in cytoskeleton organization and accumulation (Figure 1), being Dupuytren fibroblasts the ones showing thicker and more accumulation of actin fibers in the cell body (Figure 8C). This cytoskeleton organization of Dupuytren fibroblasts is related to myofibroblast phenotype, which are differentiated fibroblasts found in wound healing and present an increased contractile activity. Many cell types can differentiate into myofibroblasts, such as smooth-muscle cells, cardiomyocytes, bone marrow cells, etc. Nevertheless, the vast majority of myofibroblasts come from neighboring fibroblasts. The mechanical properties of these fibroblasts had already been measured in a previous study [7]; however, they used non-calibrated cantilevers (MLCTs), where (unavoidable) errors in the cantilever calibration could induce misleading values. Therefore, in this study, we used PFQNM cantilevers, which are pre-calibrated, to minimize errors. Besides, they calculated the elastic modulus and dynamic viscosity using a stress relaxation methodology, but here we employed oscillatory testing, which is a more robust approach to determine the viscoelastic properties of cells. We measured the complex elastic modulus (E*) using AFM over three frequency decades (1–1000 Hz); however, analyses were performed only from 1 Hz to 100 Hz due to the limited response time of the z-piezo. The E′ data at 1 Hz were around 3 kPa for healthy and scar fibroblasts, and 4 kPa for Dupuytren fibroblasts (Table 1) and all of them showed the same tendency: they increased weakly with frequency following a power law with an exponent around 0.1. The loss modulus E″ was around 1/6 of the storage modulus E′ in all cell types and increased similarly up to 10 Hz, but it presented a greater frequency dependence at higher frequencies (Figure 2). After viscous drag correction (cantilever-liquid hydrodynamics) [25], the loss modulus still presented a more marked frequency dependence, which is intrinsic to cell viscoelastic behavior (Figure S19). The hydrodynamic correction factor b(0) obtained for the PFQNM cantilever is relatively small in comparison to other cantilevers due to their small dimensions and large stiffness [24]. The values of the loss tangent are close to 0.2, and power-law exponents are around 0.1 (Table 1), which is in agreement with previous works found in the literature [26,27,28]. Larger differences in cell’s loss tangent could have been expected due to the different cytoskeleton organization; however, as fibroblasts do have already a well-developed cytoskeleton network, especially on hard substrates such as Petri dishes, the formation of few extra fibers in Dupuytren fibroblasts’ cytoskeleton does not result in measurable changes in loss tangent. The loss tangent is rather a useful rheological parameter for measuring cytoskeleton changes after drug treatment or distinguishing between different cell lines.

Over the last couple of years, evidence has accumulated that proposes the existence of 1 or 2 power-law regimes of cytoskeleton dynamics at high frequencies [29,30]. In the early days, most of the rheological measurements on cells were performed up to a limited oscillatory frequency of 100–200 Hz [25,26]. Active and passive microrheology have shown that E′ and E″ are coupled following a weak power-law with an exponent around 0.05–0.35 [26,31]. This behavior was interpreted in terms of soft glassy rheology theory (SGR) and suggested that cells with an exponent near zero resembled a soft glassy material close to the glass transition, which is also the case for the three fibroblasts studied in this work [32]. Exponent values of 0.1 suggest that the three measured fibroblast types resemble a soft glassy material close to the glass transition. Recently, cell rheological experiments increasing the frequency regime over 5–6 decades from 0.01 Hz to 1 kHz have been performed. In these measurements, a second regime at higher frequencies (100 Hz–1 kHz) is visualized with a second power-law exponent around 0.3–1 attributed to single filaments dynamics [29,33]. Our AFM rheological experiments cover only frequencies up to 100 Hz; thus, we are not able to observe the second regime because of frequency limitations. Rigato et al. used the high-speed AFM to study the rheological behavior of different cell types under cytoskeletal drug treatments spanning a high-frequency range (1 Hz–100 kHz). They saw two dynamic regimes, one at low frequencies (1 Hz–1 kHz) and another at high frequencies (1 kHz–100 kHz) [29]. Our experimental design reached frequencies up to 1 kHz; however, due to the limited response of the piezo, data were just analyzed until 100 Hz, showing only one power-law exponent. Therefore, future experiments employing faster AFMs encompassing higher frequency ranges would provide data on cell behavior at shorter time scales.

### 3.2. ML-7 Effect on Cell Mechanics

**Figure 9:**
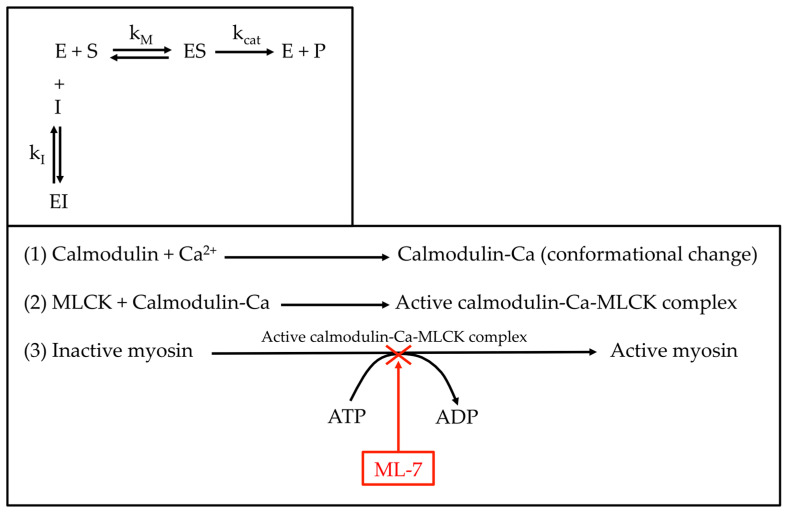
Schematic mechanism of a competitive inhibition and ML-7 place of action.

The organization of actin and myosin II mediates many cellular processes, such as cell migration, division, adhesion, etc. [34,35]. Cell contraction requires actin–myosin binding and sliding along actin filaments that depends on the phosphorylation of the myosin regulatory light chain (MRLC). The phosphorylation of myosin II at its regulatory light chain is mediated through the MLCK enzyme, which is a Ca^2+^/calmodulin-dependent enzyme [16]. The activation of MRLC facilitates actin (β-actin and α-actin in myofibroblasts) and non-muscle myosin binding. Non-muscle myosin is believed to be a key protein for cell contraction and a regulator in wound and scar contraction [36]. When intracellular calcium increases, calcium interacts with calmodulin, which at the same time binds to MLCK. This calcium-calmodulin-MLCK complex formation activates MLCK enzymatic activity. MLCK activity is inhibited by drugs such as naphthalene sulfonamide derivatives. ML-7 is a member of naphthalene sulfonamide derivatives that acts by binding directly to MLCK on its active site (Figure 9). It is a reversible ATP-competitive inhibitor, and when ML-7 binds to MLCK, it blocks for a limited time the binding of ATP to the enzyme’s active site, therefore preventing the phosphorylation of myosin RLC, non-muscle myosin activation, which leads to inhibition of actin–myosin binding and cell contraction. It has been found that ML-7 induces apoptosis in mammary and prostate cancer cells in vitro and also stimulates the ability of a chemotherapeutic drug to prevent the growth of mammary and prostate tumors in vivo [17]. Pancreatic cancerous cells were also treated with ML-7 producing a rounding up and reduction of the number of stress fibers in the cell body [21].

Although it is not clear if MLCK is overexpressed in myofibroblasts, an increase of MLCK expression has been observed in myofibroblasts obtained from the nodules of the palm of Dupuytren’s patients in comparison to normal and cords fibroblasts [37,38]. With these findings, we propose that the inhibition of MLCK by ML-7 is a potential target to reduce myofibroblasts contractility.

ML-7 effect is dose-dependent, from provoking no reaction at low concentrations to cell death at higher doses [22]. 3T3 and NRK cells subjected to ML-7 exhibited a retraction of lamellipodia and softening of the cells; however, in some cases cell recovery was seen over an hour.

In this study, we treated the three fibroblast types with two different concentrations of ML-7. We used 1 and 3 µM as intermediate concentrations to assess differences in cell behavior among the cell types. Dupuytren fibroblasts differ from their healthy counterparts as they contain extra actin fibers, α-SMA, that result in a more-developed cytoskeleton network. This is translated into more elastic filaments in the cell body, more cortical tension and higher stiffness. This is in agreement with the data shown in Figure 1. Our data showed that Dupuytren fibroblasts present a more drastic reaction to the drug, and when treated with 1 µM of the inhibitor, ML-7 inhibits MLCK activity, thus resulting in disruption of the cytoskeleton network and reduction in stress fiber strength that is at the same time translated into a decrease in storage modulus and an increase in loss tangent as the cell becomes more liquid-like (Figure 6). In the case of healthy and scar fibroblasts at the same ML-7 dose, there were no visible changes in elasticity after adding the inhibitor. The different behavior of the three fibroblasts to the drug demonstrates the different nature of the cells and their different susceptibility towards MLCK inhibition. Lyapunova et al. found that the ML-7 effect on cancerous thyroid cells was less notorious than healthy counterparts [39]. In the literature, compounds from the ML-7 family, such as ML-9, have also been tested as MLCK inhibitors. Both ML-9 and ML-7 are naphthalene sulfonamide derivatives, but ML-7 inhibition is more than 30-fold more potent than that ML-9 [20,40]. These inhibitors were also used on mouse lung carcinoma cells to study their effect on the cell attachment to fibronectin substrates [41]. They suggested that MLCK phosphorylation is involved in cytoskeleton rearrangements required for the cell surface fibronectin receptor to cluster in order to achieve cell attachment to fibronectin substrates.

Myofibroblasts develop very mature focal adhesion complexes that connect the cell to the ECM and thus allow the propagation of mechanical stimuli. Focal adhesion complexes are composed of many intracellular proteins that connect the cell actin–myosin network to the ECM via integrins. Integrins are provided by extracellular, transmembrane and intracellular domains that enable the cell-ECM interplay. Changes in actin–myosin strength, such as cytoskeleton disruption through MLCK inhibition, have been seen to disentangle focal adhesion complexes leading to cell-ECM attachment interruption [42]. Our study is in agreement with the previous idea, in which MLCK-inhibited fibroblasts lose their attachment to the substrate and start floating. Therefore, it seems likely that MLCK reduces actin–myosin contraction provoking a downstream signaling pathway in which focal adhesion complexes are also inhibited, weakening cell-ECM interaction.

An increased ML-7 concentration produces a stronger effect on the mechanical properties of all cell types. Nonetheless, healthy and scar fibroblasts still showed less effect than Dupuytren fibroblasts. If cells did not present any shape or mechanical change, this does not necessarily imply that the cells have not reacted to the inhibitor in a biochemical way. The concentrations of the inhibitor used may produce effects that we are not able to pick up with the methods employed in this study. Another possible explanation is that cells in different stages of the cell cycle may show different susceptibility to the drug. Besides, since ML-7 is a reversible inhibitor, it may also happen that when the ATP concentration is greater than ML-7, ATP will preferentially bind to the enzyme restoring MLCK enzyme catalytic activity. As a matter of fact, we hypothesize that this may happen when fibroblasts start recovering their mechanical properties after cell softening. When cells experience a drastic change in shape and mechanical properties, it is often difficult to overcome the inhibition process and they end up dying. Cells experiencing death, in addition to cell shrinkage (acquiring a rounded shape), the formation of blebs and protrusions in the cell membrane, as well as fragmentation, is observed, where all these processes can be seen at different stages of apoptosis.

Our data support the notion that MLCK inhibition plays a role in fibroblasts and especially in myofibroblasts contraction. The more drastic effect of Dupuytren fibroblasts on ML-7 treatment supports its myofibroblasts phenotype and the potential use of ML-7 as a therapeutic treatment for Dupuytren’s disease. Although our data show promising results, future studies are needed to corroborate our findings and to encompass different approaches, for example, looking at calcium-independent pathways (Rho-kinase), which are also known to play an important role in myofibroblasts contraction.

## 4. Materials and Methods

### 4.1. General Materials

ML-7 inhibitor was purchased from Merck (Sigma-Aldrich, St. Louis, MO, USA, Merck, Rahway, NJ, USA, 475880). A 50 µM stock solution was prepared in DMSO. Further dilutions were made in complete cell culture medium.

### 4.2. Cell Isolation and Cell Culture

Primary fibroblasts were isolated from 3 surgically removed skin tissues of a 55-year-old female patient’s left-hand palmar fascia regions. Scar fibroblasts were extracted from the dermal scar excision and healthy fibroblasts from the adjacent skin tissue. Dupuytren fibroblasts were isolated from nodules on the palmar fascia. The protocol to isolate the three cell types has been described previously [7]. All fibroblasts were cultured with DMEM (Sigma-Aldrich, FG0435) medium supplemented with 10% fetal bovine serum (Sigma-Aldrich, F7524) and 2% penicillin-streptomycin (Sigma-Aldrich, P0781) and incubated at 37 °C in a 5 % CO_2_ humidified chamber. Cells were seeded in Petri dishes 48 h prior to AFM experiments. Passages from 4 to 10 were used for the measurements.

### 4.3. AFM Experiments

AFM measurements were performed with an MFP3D AFM (Asylum Research, Santa Barbara, CA, USA) combined with an optical microscope (Zeiss Axiovert 135, Zeiss, Oberkochen, Germany) to be able to control tips and samples. Petri dishes, containing samples, were fixed to an aluminum holder with vacuum grease and mounted on the AFM stage with two magnets. The entire set-up was enclosed in a home-built polymethylmethacrylate (PMMA) box in order to inject and maintain 5% CO_2_ during experiments. Fibroblasts were probed 48 h after seeding and data were collected on the nuclear region.

In this work, two types of experiments were carried out. Firstly, rheological properties of the cells were measured. For such purpose, PFQNM-LC-A-CAL cantilevers were employed (Bruker, Karlsruhe, Germany, pre-calibrated spring constant around 100 pN/nm and 45 kHz resonance frequency in air), which have a three-sided pyramidal tip (15–25° opening angle) with a protrusion of 75 nm tip radius and 0.8–1 µm length at its end. Around 90 cells of each type were probed and the apparent Young’s modulus values were extracted from regular approach curves. In addition, sweep frequency force curves were employed to obtain individually the elastic and viscous properties. Shortly, in sweep frequency curves a sinusoidal modulation in z-height with increasing frequency (from 1 Hz to 1 kHz) is applied for 8.7 s after the trigger threshold was achieved. After 1 s, the sinusoidal modulation was started while still in contact with the cell and after an extra 1 s this step was reversed (Figures S1–S3). From these experiments, storage and loss modulus, loss tangent and power-law exponent parameters were acquired. Each force map was spaced 5 × 5 μm and composed of 16 or 256 force curves (4 × 4 or 16 × 16 lines per frame). Typically, force curves were recorded at a scan rate of 2 Hz, corresponding to a maximum velocity of 20 µm/s.

For the second set of experiments, cells’ mechanical properties were assessed before and after ML-7 addition for each fibroblast. Pre-calibrated cantilevers (MLCT-SPH-DC-E, Bruker, nominal spring constant 150 pN/nm and 17 kHz resonance frequency in air) with a semi-spherical tip of 5.5-µm radius were used to assess cell properties. At least 20 fibroblasts were measured for each cell type. Concentrations of 1 and 3 µM ML-7 were used in order to assess differences in cell behavior. Each force map was spaced 5 × 5 μm and composed of 4 or 36 force curves (2 × 2 or 6 × 6 lines per frame). Force curves were recorded at a scan rate of 1.3 Hz, corresponding to a maximum velocity of 10.4 µm/s. Indentation depths were always greater than 500 nm in order to average the stiffness over a large contact area, which gives values that do not depend on local variations of the cytoskeleton structure.

### 4.4. AFM Data Analysis

The data analysis software IGOR 7 (Wavemetrics, Lake Oswego, OR, USA) was used to evaluate the mechanical properties of cells in terms of Young’s or elastic modulus (E). The Hertzian model for spherical/parabolic tips was used to calculate the apparent Young’s modulus for each force curve within a force map. The logarithmic histogram and median with 25/75 percentiles of Young’s modulus were considered representative modulus of each force map. Sweep frequency data were fitted with the power-law structural damping model [25]. E* data are separated into real (in phase) and imaginary (out of phase) parts. The real part represents the storage modulus, and it is a measure of the elastic energy stored and recovered per cycle of oscillation. The imaginary part depicts the loss modulus, and it accounts for the energy dissipated per cycle of sinusoidal deformation. We also calculate the loss tangent, which is an index of the solid-like (<1) or the liquid-like (>1) behavior of the cell. This model assumes a storage modulus that increases with the frequency following a power law with exponent *α* and a loss modulus that includes a term that is a fraction *η* of the storage modulus and a Newtonian viscous term (Supplementary, sweep frequency).

### 4.5. Immunostaining

Forty-eight hours after seeding of cells on Petri dishes, cells were fixed with 4% paraformaldehyde for 15 min and permeabilized with 0.1% Triton ×100 for 5 min. Samples were washed with PBS after each step and then incubated with ActinGreen 488 ReadyProbes Reagent (Invitrogen, ThermoFisher, Waltham, MA, USA, R37110) (2 drops in 1 mL PBS) for F-actin staining for 30 min at room temperature. Finally, cells were stored in PBS at 4 °C prior to image acquisition. Nikon Eclipse Ti inverted epi-fluorescence microscope (Nikon Instruments Inc., Melville, NY, USA) with 40× objective lens was used to observe cells and collect fluorescent images.

### 4.6. Statistical Analysis

Differences between apparent Young’s modulus values obtained for each cell type were checked for statistical significance with the Wilcoxon signed-rank test and for effect size with Cohen’s d test using IGOR. For each force volume the median of E values was calculated. The effect size (Cohen’s d) between the means of E and the significance of difference (Wilcoxon signed-rank) between the medians of the E for each type of cell was tested. For *p*-values obtained from the Wilcoxon signed-rank test, * indicating *p* < 0.01 and Cohen’s d test with # indicating 0.2 < d < 0.5 and ## indicating 0.5 < d < 1.0.

## 5. Conclusions

We have assessed the rheological properties of three primary fibroblasts, all established from the palm of the same patient. Dupuytren fibroblasts, expressing a myofibroblast phenotype, presented larger apparent Young’s modulus values than healthy or scar fibroblasts, which is attributed to extra actin fibers. We have also determined the effect of myosin II function on the stiffness of fibroblasts. ML-7, an MLCK inhibitor, was used to deactivate myosin II in these three fibroblast types. The changes in rheological properties were measured by AFM employing oscillatory testing as a methodology to extract separately elastic and viscous contributions. In summary, it was shown that myosin activation is a key element for cell contractility, and ML-7 reduces fibroblasts and myofibroblasts contractility through the inhibition of MLCK, especially Dupuytren fibroblasts, which were the most susceptible. ML-7 effect suggests its potential use as a pharmacological treatment for Dupuytren’s disease.

## Figures and Tables

**Table 1:** Apparent Young’s modulus, storage and loss modulus at 1 Hz, loss tangent at 1 Hz and power-law exponent median values for each cell type (*n* = 90).

	Apparent Young’s Modulus (Pa)	Storage Modulus (Pa)	Loss Modulus (Pa)	Loss Tangent	Power Law Exponent
Healthy Fib.	3345	3260	473	0.18	0.11
Scar Fib.	3940	3024	576	0.19	0.13
Dupuytren Fib.	5364	4260	742	0.18	0.12

**Table 2:** Percentage of fibroblasts that die, recover or experience no change after addition of 1 or 3 µM of ML-7 inhibitor.

	Healthy Fibroblasts	Scar Fibroblasts	Dupuytren Fibroblasts
1 µM ML-7 (*n* = 15)	Dead: 20%	Dead: 0%	Dead: 13%
	Recovery: 20%	Recovery: 20%	Recovery: 47%
	No change: 60%	No change: 80%	No change: 40%
3 µM ML-7 (*n* = 20)	Dead: 39%	Dead: 39%	Dead: 60%
	Recovery: 34%	Recovery: 25%	Recovery: 10%
	No change: 27%	No change: 36%	No change: 30%

## Data Availability

Part of the data generated and/or analyzed during the current study is available in zenodo (https://doi.org/10.5281/zenodo.7376914).

## Supplementary Materials

The supporting information can be downloaded at: https://www.mdpi.com/article/10.3390/ijms24032043/s1.
