# Supplementary material for: Comparison of Rheological Properties of Healthy versus Dupuytren Fibroblasts When Treated with a Cell Contraction Inhibitor by Atomic Force Microscope
Source: Int J Mol Sci. 2023 Jan 20;24(3):2043. doi: 10.3390/ijms24032043 (PMC9917339; doi:10.3390/ijms24032043)
Supplement: Supplementary file 1 [file ijms-24-02043-s001.zip › ijms-2097541-supplementary.pdf]

Supporting material

AFM original data are obtained as follows, deflection versus z-piezo movement (d- z):

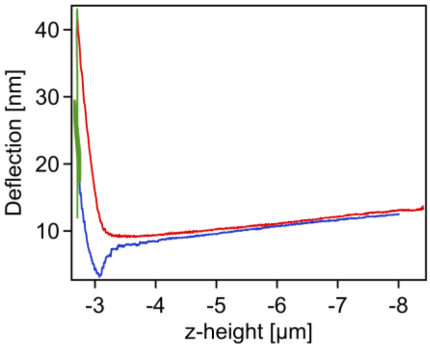

Figure S1. Example of a sweep modulation force curve. Deflection versus z-height display. The approach curve is in red, the retract curve in blue and the dwell in green (sweep modulation). The hysteresis between approach and retract curve is due to the cell's viscous contribution and adhesion.

However, to compute Young's modulus E, which is a measure of the sample's elasticity, force curves need to be converted into force versus indentation (F- δ) data. For that, the total force applied to the sample can be calculated using Hooke's law:

$$F = k \cdot (d - d_0)$$

where k is the spring constant of the cantilever and d the cantilever deflection.
Indentation data can be derived from the following expression:

$$\delta = (z - z_0) - (d - d_0)$$

where z_0 is the cantilever displacement at the tip-sample contact point and d_0 is the deflection offset. To obtain E, (F- δ) curves are analyzed with the appropriate contact model. In our experiments we used Hertz's model for spherical tips (more exactly: for parabolic tips, which are a good approximation and commonly used for spherical tips):

$$F = \frac{4}{3}\frac{E}{(1 - \nu^2)}\sqrt{R}\delta^{3/2}$$

where R is the radius of curvature in spherical/paraboilic probes and ν is the Poisson's ratio (is assumed to be 0.5).

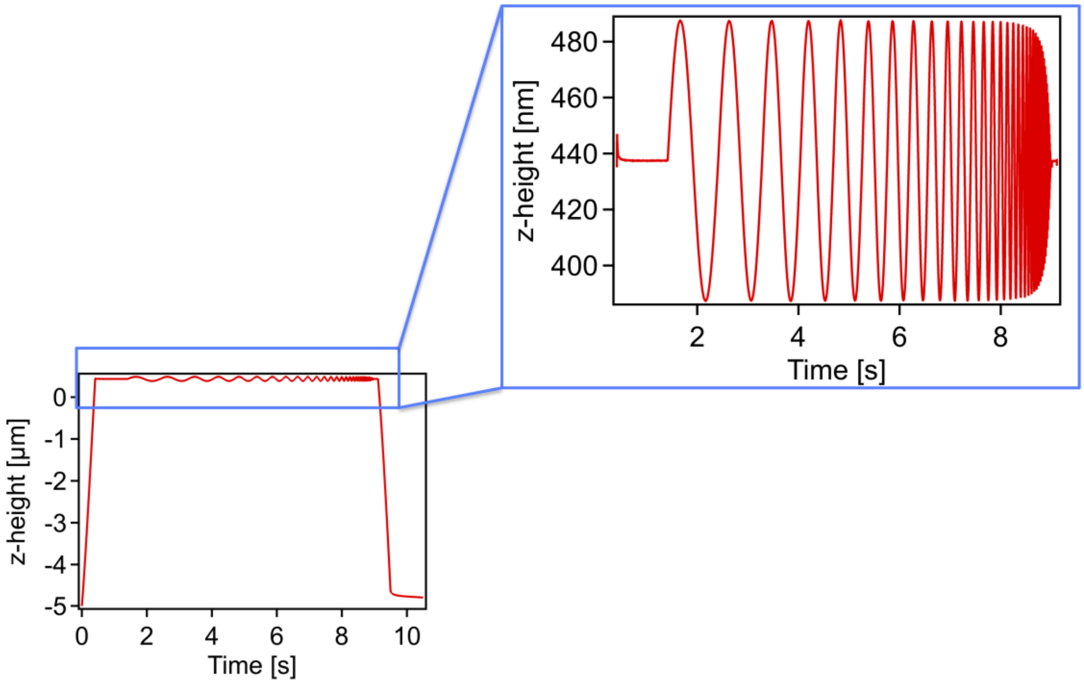

Figure S2. Z-height versus time in sweep modulation. The z-height reflects the piezo movement in z direction over time while the sweep modulation is taking place. The blue box zooms in the sweep modulation part.

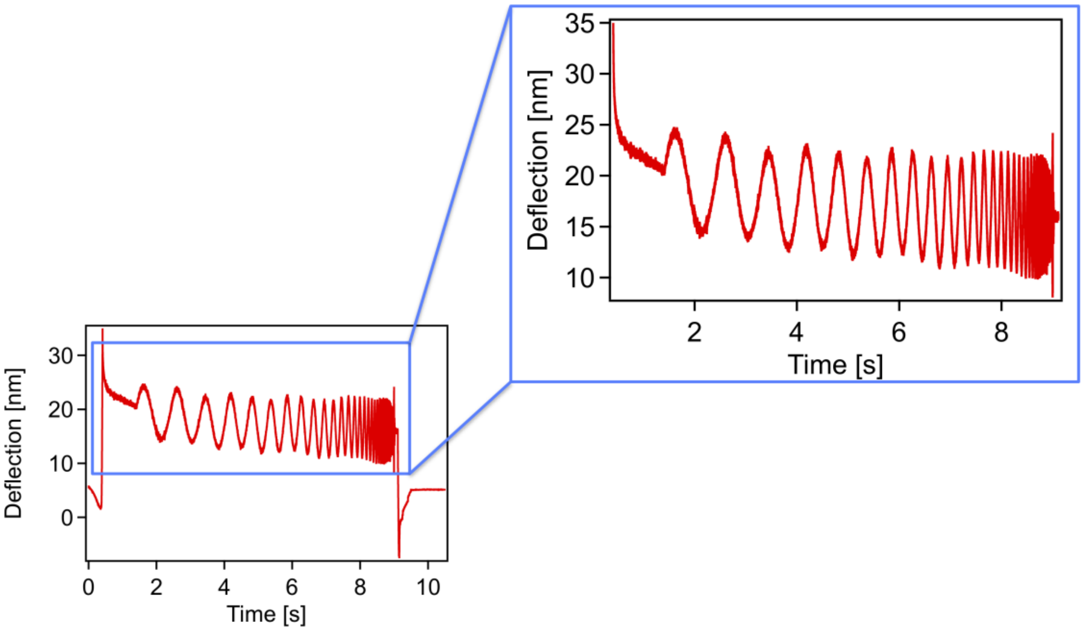

Figure S3. Deflection versus time display during sweep modulation. The deflection reflects the cantilever movement over time while the sweep modulation is taking place in a force curve. The blue box zooms in the sweep modulation part.

In sweep modulation methodology a sinusoidal modulation with increasing frequency is added to the z-piezo while the tip is in contact. Typically, we use a dwell time of 8.7 seconds, where during the first second no modulation is applied since the cell creeps substantially. Then, the modulation starts and the frequency is swept from 1Hz to 1kHz, where for each frequency only one cycle is applied. The frequency series is designed as a geometric series, so

that in each decade we use the same number of frequencies. Typically, the frequency increases by a factor of 1.15 from cycle to cycle, which results in 17 frequency values per decade, which are equally spaced on a logarithmic scale.

We employed the power law structural damping model as has been introduced to AFM rheology by (Alcaraz *et al.*, 2003). Here, the complex modulus G^* is written as:

$$G^*_{(\omega)} = G_0 * (1 + i\eta) * \left(\frac{\omega}{\omega_0}\right)^{\alpha} + \mathrm{i}\,\mu\,\frac{\omega}{\omega_0}$$

where G_0 is the absolute value of the moduli, i is the imaginary unit, η is the ratio between loss modulus and storage modulus (often called the loss tangent), ω_0 is the frequency scale (in our case we use 1 Hz), α is the power law exponent of the sample, and μ is the strength of the hydrodynamic damping, which will depend on the shape of the cantilever and the viscosity of the medium. The real part represents the storage modulus and the imaginary part corresponds to the apparent loss modulus, which has two contributions; (1) from the sample, which scales with the same power law exponent as the storage modulus, and the hydrodynamics, which is directly proportional to the frequency. Thus, we can first obtain the power law exponent by fitting the storage modulus as a function of frequency, and then in a second step obtain the loss tangent η and the strength of the hydrodynamic damping of the cantilever μ.

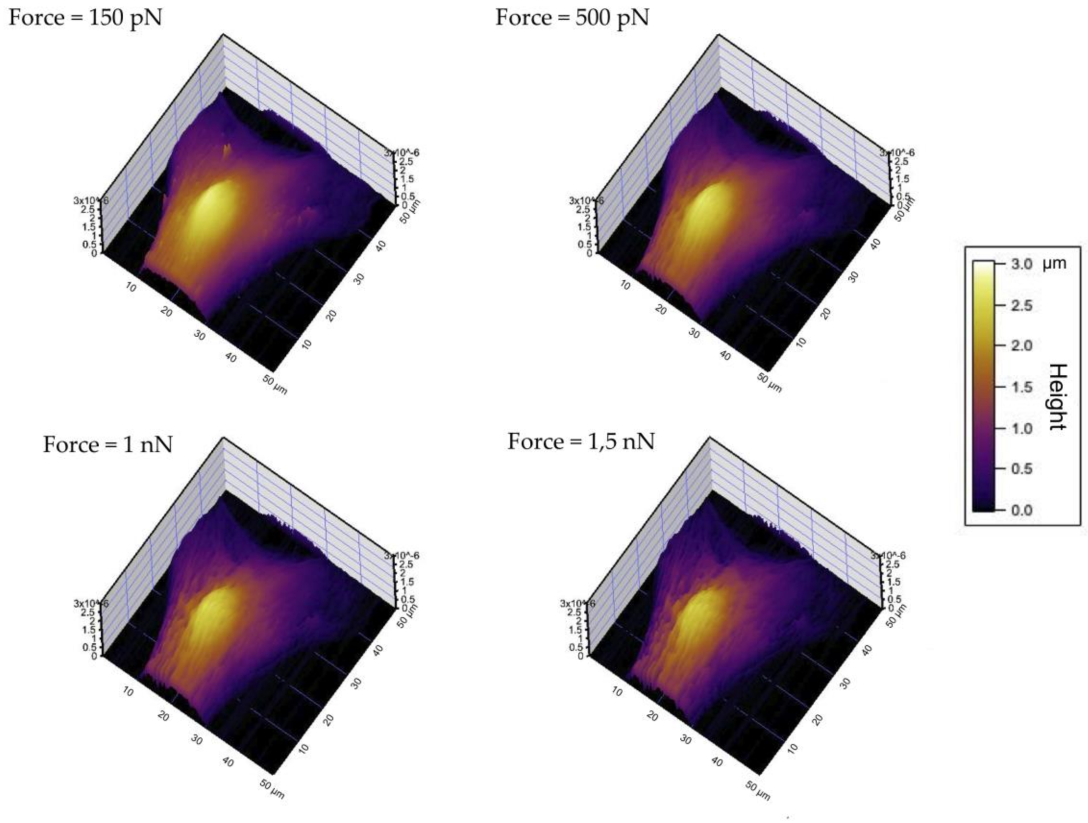

(a)

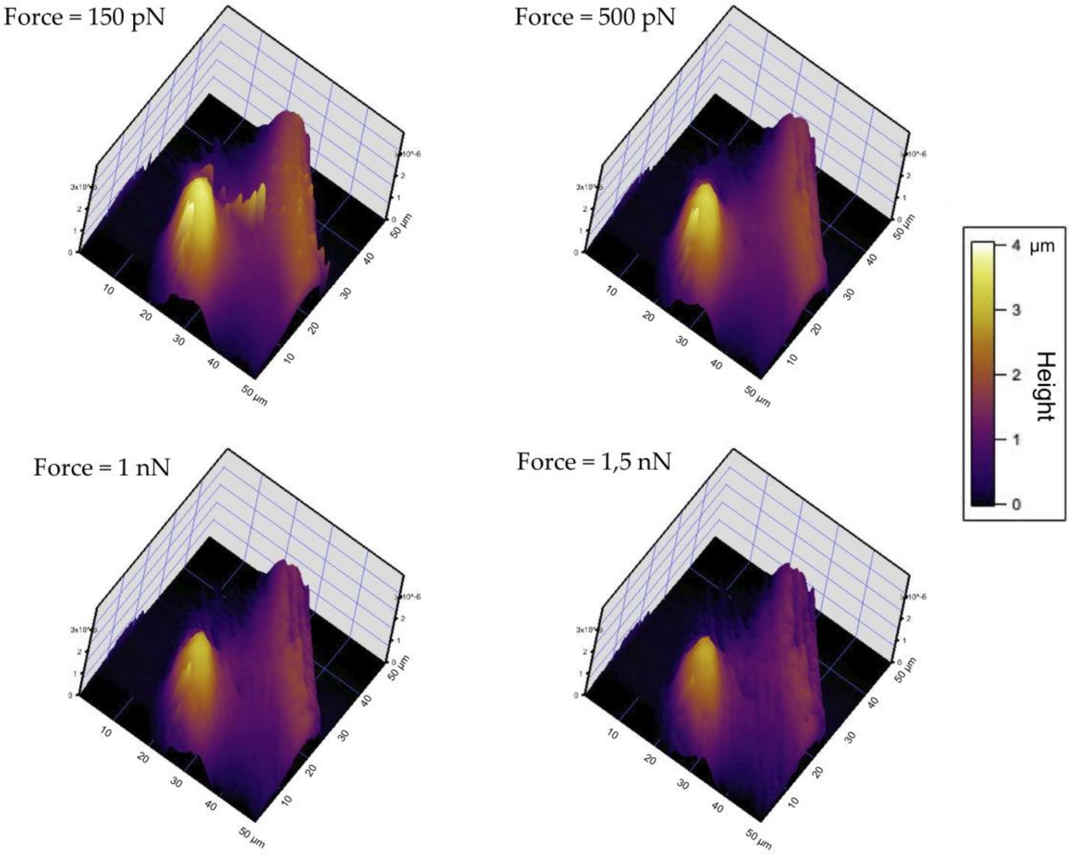

Force = 150 pN
Force = 500 pN
Force = 1 nN
Force = 1,5 nN
50 µm
4 µm
Height

(b)

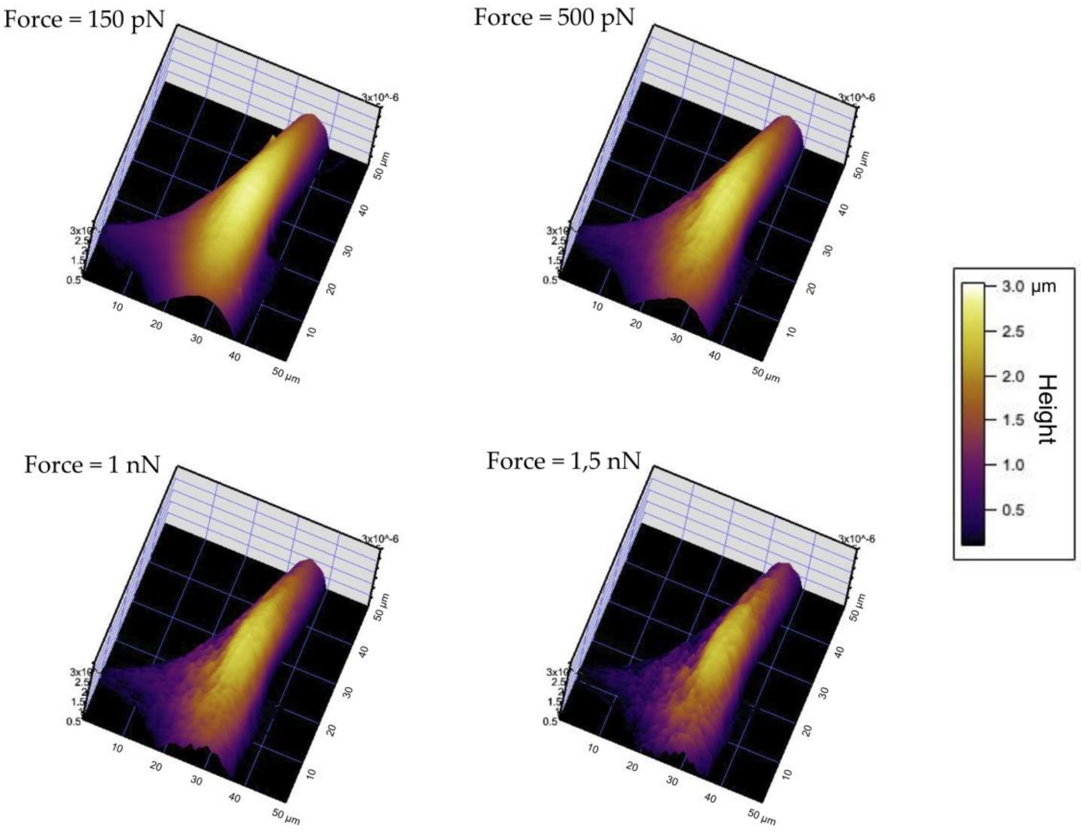

(c)

Figure S4. 3D-images representation of a) Healthy, b) scar and c) Dupuytren fibroblasts obtained from high-resolution JPK force maps. Forces maps of 50x50 µm with pixel resolution of 135x135. The color bar indicates the cell height.

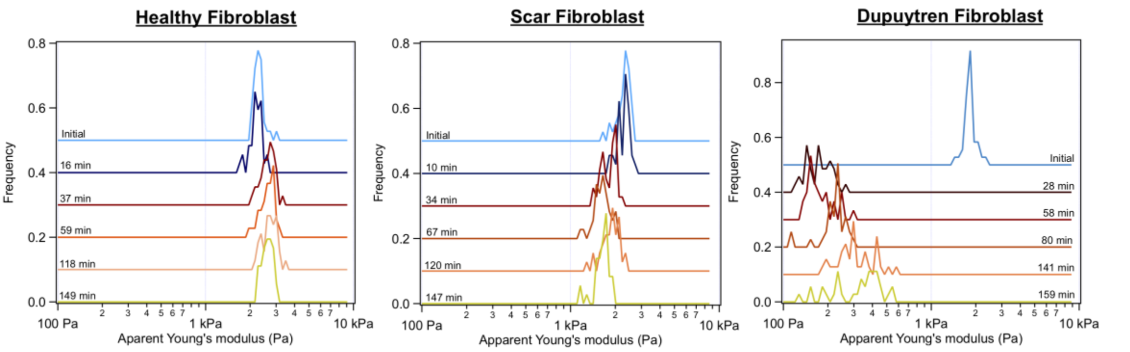

Figure S5. Histogram distribution of the Apparent Young's modulus of each fibroblast before and after 1 µM ML-7 addition (n = 15).

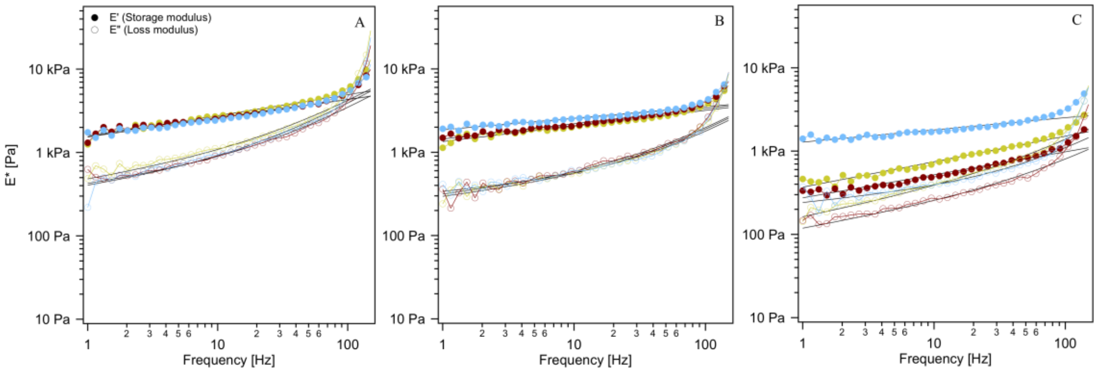

Figure S6. Frequency dependence of the storage (filled symbols) and loss modulus (open symbols) before and after addition of 1 μM ML-7 in a) healthy fibroblast (blue: initial; red: 37 min after ML-7; yellow: 149 min after ML-7), b) scar fibroblast (blue: initial; red: 34 min after ML-7; yellow: 147 min after ML-7) and c) Dupuytren fibroblast (blue: initial; red: 58 min after ML-7; yellow: 159 min after ML-7). Solid black lines are the fit of the power law structural damping model. ML-7 produces a drop in storage and loss modulus in C (n = 15).

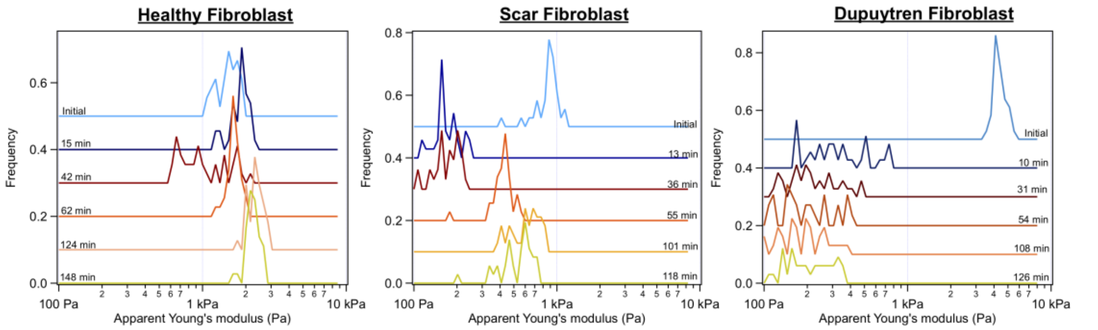

Figure S7. Histogram distribution of the Apparent Young's modulus of each fibroblast before and after 3 μM ML-7 addition (N = 20).

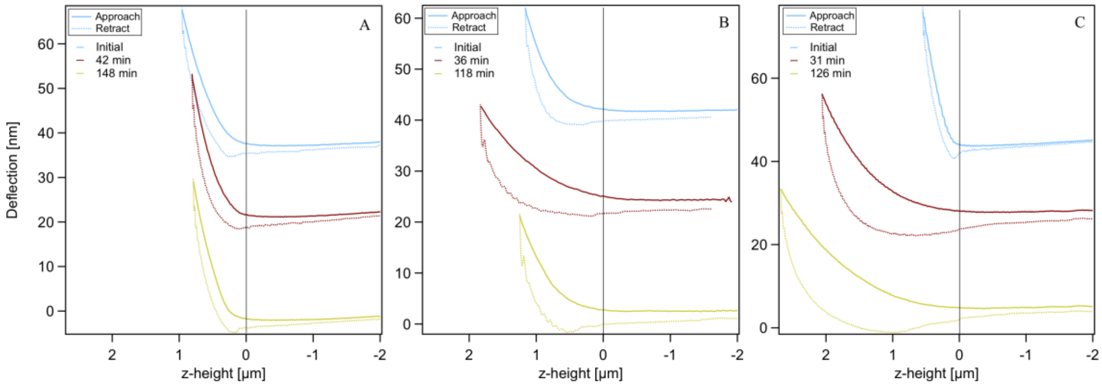

Figure S8. Example of a regular force curve of A) healthy, B) scar and C) Dupuytren fibroblast before and after 3 μM of ML-7 addition. Solid line is the approach curve and the dashed line retract curve. The effect of ML-7 in the cell is visible in B and C panels with a decrease in the slope and an increase in the hysteresis between the approach and retract curve. The increment in the hysteresis reflects an increase in the viscosity of the cell due to cell cytoskeleton disruption. The B panel also reflects a cell recovery after 118 minutes of ML-7 addition, the slope of the curve (yellow) increases with respect to the red curve.

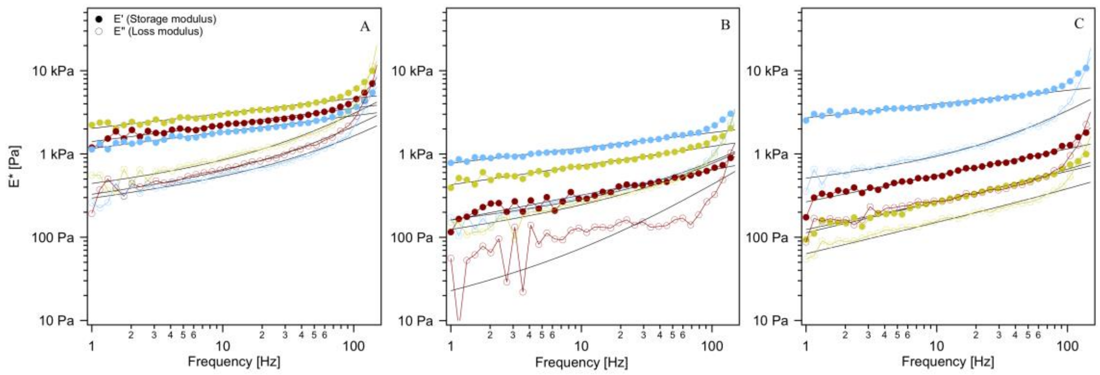

Figure S9. Frequency dependence of the storage (filled symbols) and loss modulus (open symbols) before and after addition of 3 µM ML-7 in a) healthy fibroblast (blue: initial; red: 42 min after ML-7; yellow: 148 min after ML-7), b) scar fibroblast (blue: initial; red: 36 min after ML-7; yellow: 118 min after ML-7) and c) Dupuytren fibroblast (blue: initial; red: 31 min after ML-7; yellow: 126 min after ML-7). Solid black lines are the fit of the power law structural damping model. ML-7 produces a drop in storage and loss modulus in B and C panels (n = 20).

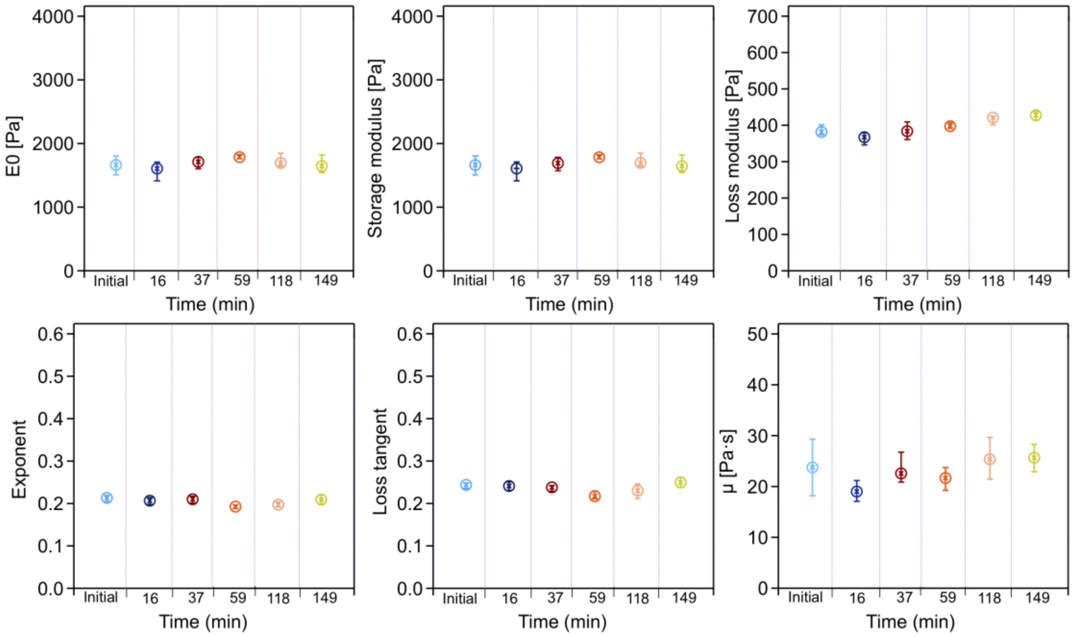

Figure S10. Median with 25/75 percentiles representation of rheological properties of a healthy fibroblast before and after 1 µM ML-7 addition. From left to right: scale factor for storage and loss moduli, storage modulus, loss modulus, power law exponent, loss tangent and Newtonian viscous term (n = 15).

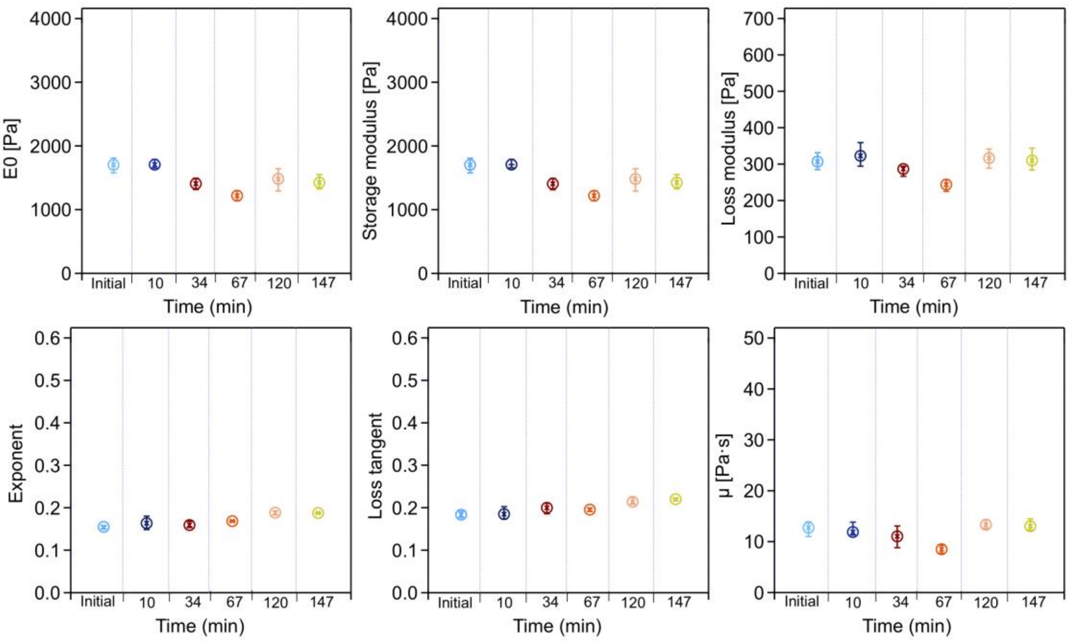

Figure S11. Median with 25/75 percentiles representation of rheological properties of a scar fibroblast before and after 1 µM ML-7 addition. From left to right: From left to right: scale factor for storage and loss moduli, storage modulus, loss modulus, power law exponent, loss tangent and Newtonian viscous term (n = 15).

.

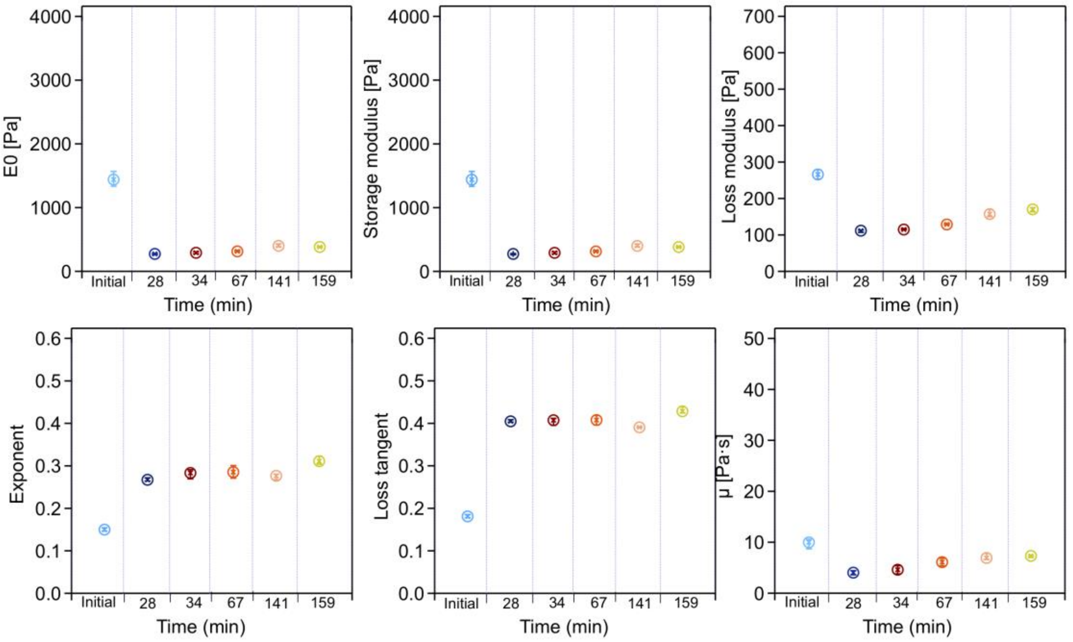

Figure S12. Median with 25/75 percentiles representation of rheological properties of a Dupuytren fibroblast before and after 1 µM ML-7 addition. From left to right: From left to right: scale factor for storage and loss moduli, storage modulus, loss modulus, power law exponent, loss tangent and Newtonian viscous term (n = 15).

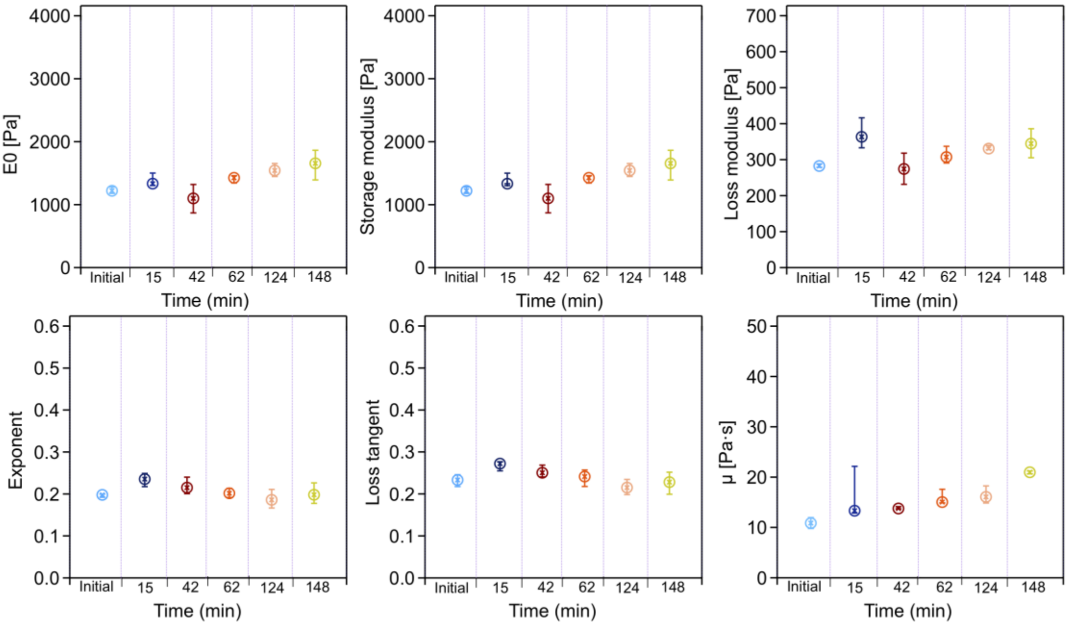

Figure S13. Median with 25/75 percentiles representation of rheological properties of a healthy fibroblast before and after 3 μM ML-7 addition. From left to right: From left to right: scale factor for storage and loss moduli, storage modulus, loss modulus, power law exponent, loss tangent and Newtonian viscous term (n = 20).

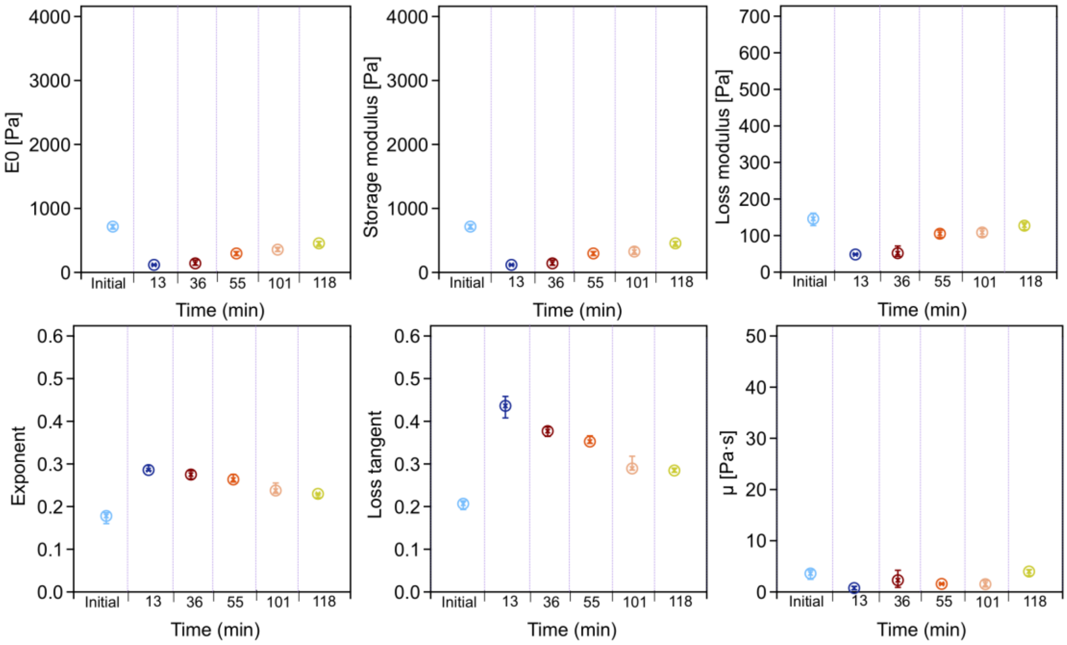

Figure S14. Median with 25/75 percentiles representation of rheological properties of a scar fibroblast before and after 3 μM ML-7 addition. From left to right: From left to right: scale factor for storage and loss moduli, storage modulus, loss modulus, power law exponent, loss tangent and Newtonian viscous term (n = 20).

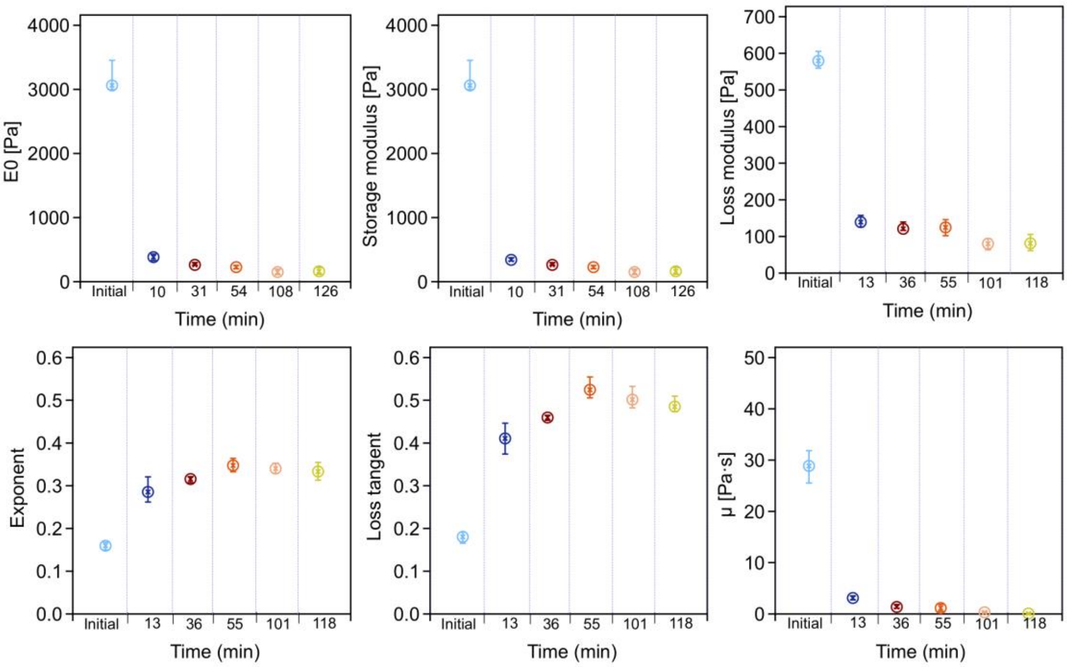

Figure S15. Median with 25/75 percentiles representation of rheological properties of Dupuytren fibroblast before and after 3 μM ML-7 addition. From left to right: From left to right: scale factor for storage and loss moduli, storage modulus, loss modulus, power law exponent, loss tangent and Newtonian viscous term (n = 20).

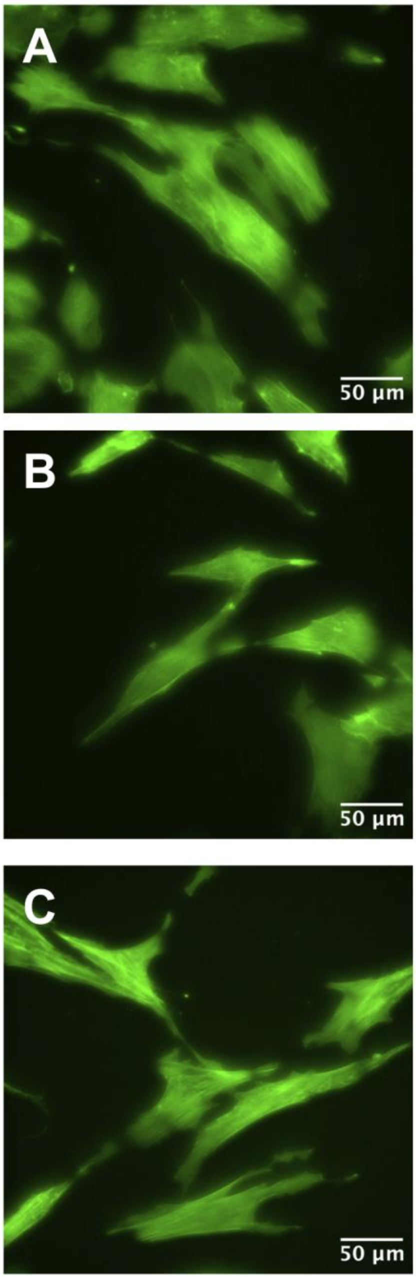

Figure S16. Actin fibers staining after DMSO addition, same concentration as ML-7 experiments. A) Healthy, B) Scar and C) Dupuytren fibroblasts. Experiments suggest that cell mechanics changes are due to ML-7 effect and not DMSO or time effect.

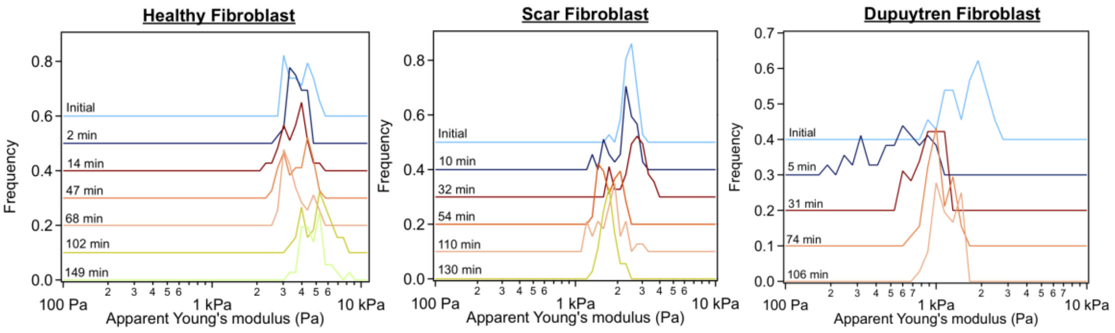

Figure S17. Histogram distribution of the Young's modulus of each type of fibroblasts with DMSO at the same concentration as ML-7 experiments. Cells were followed the same way as ML-7 experiments to verify if changes in cell stiffness were due to ML-7 or DMSO effect. Cells maintained their stiffness along the entire experiment; therefore, cell mechanical changes after ML-7 addition were due to the inhibition effect on the cell (n = 5).

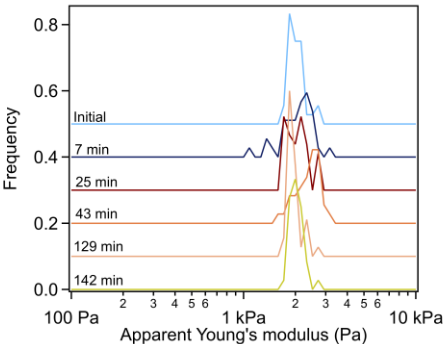

Figure S18. Histogram distribution of the Young's modulus of Dupuytren fibroblasts without ML-7 addition. Cells were followed the same way as ML-7 experiments to verify if changes in cell stiffness were due to ML-7 effect or just cell cytoskeletal changes over time. Cells maintained their stiffness along the entire experiment (n = 5).

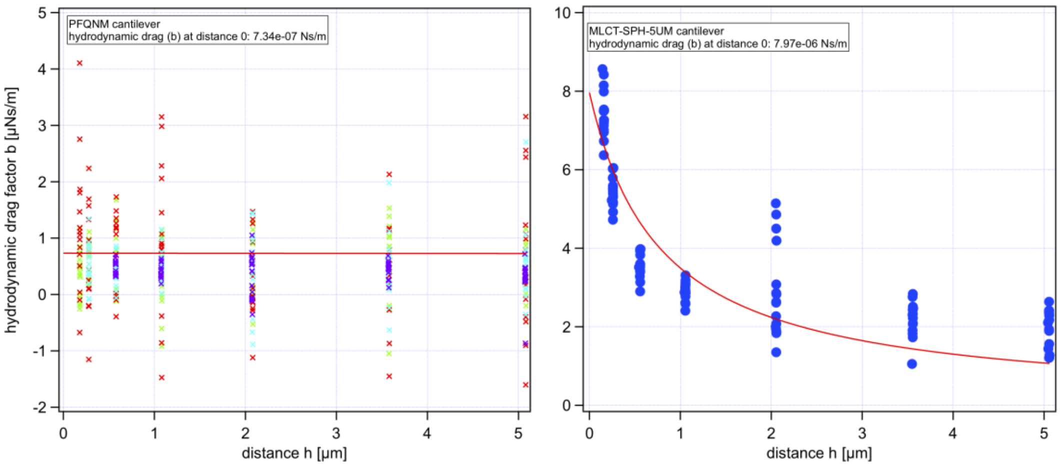

Figure S19. Drag factor at contact for PFQNM and MCLT-SPH-5UM cantilevers. PFQNM could be considered of having rectangular shape and MLCT-SPH-5UM V-shape. The b(0) was calculated measuring the drag factor at different distances from the sample as previously described[22]. The color code represents different cantilever amplitudes used (50, 100, 200, 500 nm) and the cantilever was subjected to sinusoidal modulation sweeping the frequency from 1 Hz to 100 Hz.
